# The forkhead DNA-binding domain binds specific G_2_-rich RNA sequences

**DOI:** 10.1093/nar/gkad994

**Published:** 2023-11-02

**Authors:** Caroline Zutterling, Anne-Laure Todeschini, Deborah Fourmy, Didier Busso, Xavier Veaute, Frédéric Ducongé, Reiner A Veitia

**Affiliations:** Université Paris Cité, CNRS, Institut Jacques Monod, CNRS UMR7592, Paris 75013, France; Université Paris Cité, CNRS, Institut Jacques Monod, CNRS UMR7592, Paris 75013, France; Molecular Imaging Research Center, Fontenay-aux-Roses, France; Université Paris Saclay, France; Institut de Biologie François Jacob, CEA, Fontenay aux Roses, France; Université Paris Saclay, France; Institut de Biologie François Jacob, CEA, Fontenay aux Roses, France; CIGEx platform. UMR Stabilité Génétique Cellules Souches et Radiations, Fontenay-aux-Roses, France; Université Paris Saclay, France; Institut de Biologie François Jacob, CEA, Fontenay aux Roses, France; CIGEx platform. UMR Stabilité Génétique Cellules Souches et Radiations, Fontenay-aux-Roses, France; Molecular Imaging Research Center, Fontenay-aux-Roses, France; Université Paris Saclay, France; Institut de Biologie François Jacob, CEA, Fontenay aux Roses, France; Université Paris Cité, CNRS, Institut Jacques Monod, CNRS UMR7592, Paris 75013, France; Université Paris Saclay, France; Institut de Biologie François Jacob, CEA, Fontenay aux Roses, France

## Abstract

Transcription factors contain a DNA-binding domain ensuring specific recognition of DNA target sequences. The family of forkhead (FOX) transcription factors is composed of dozens of paralogs in mammals. The forkhead domain (FHD) is a segment of about 100 amino acids that binds an A-rich DNA sequence. Using DNA and RNA PCR-SELEX, we show that recombinant FOXL2 proteins, either wild-type or carrying the oncogenic variant C134W, recognize similar DNA-binding sites. This suggests that the oncogenic variant does not alter the intrinsic sequence-specificity of FOXL2. Most importantly, we show that FOXL2 binds G_2_-rich RNA sequences whereas it virtually fails to bind similar sequences in DNA chemistry. Interestingly, a statistically significant subset of genes responding to the knock-down of *FOXL2/Foxl2* harbor such G_2_-rich sequences and are involved in crucial signaling pathways and cellular processes. In addition, we show that FOXA1, FOXO3a and chimeric FOXL2 proteins containing the FHD of the former are also able to interact with some of the preferred FOXL2-binding sequences. Our results point to an unexpected and novel characteristic of the forkhead domain, the biological relevance of which remains to be explored.

## Graphical Abstract

**Graphical Abstract:**
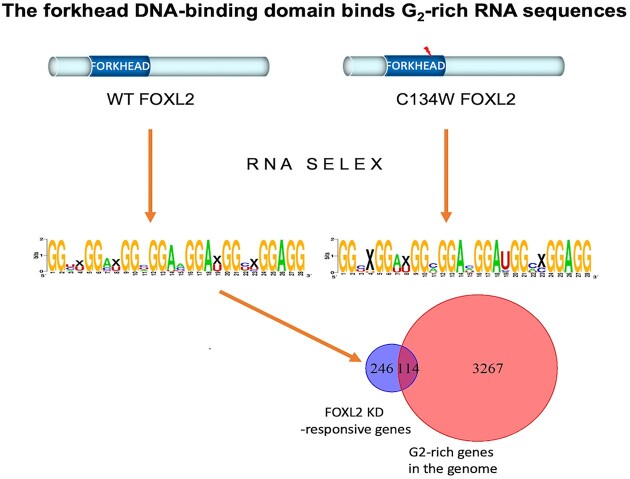

## Introduction

Transcription factors (TFs) possess DNA-binding domains (DBD) that recognize DNA target sequences in the genome. The majority of TFs recognize rather small DNA motifs, often typical of the family or subfamily to which they belong (1). The family of forkhead transcription factors (called FOX factors below) is composed of about 50 factors in humans and 44 in mice (2). These TFs are involved in a large number of biological processes, in a wide variety of cell types and share a DBD belonging to the helix-loop-helix type, called forkhead domain (FHD). The FHD is composed of a segment of about 100 amino acids folded into a winged helix pattern (3). FOXL2 is a member of the FOX family. Its germline pathogenic variants are responsible for the blepharophimosis ptosis epicanthus inversus syndrome (4), characterized by mild craniofacial defects, often associated with primary ovarian insufficiency (4,5). The protein sequence of FOXL2 is highly conserved in vertebrates and, in addition to the FHD, it contains a characteristic polyalanine (polyAla) tract of 14 residues in eutherian mammals (2). The expansion of this polyAla tract from 14 to 24 residues represents about one third of the intragenic pathogenic variations and leads to protein aggregation, both in the nucleus and in the cytoplasm, and to partial cytoplasmic mislocalization (6). In 2009, an RNAseq-based study identified the recurrent c.402C > G somatic variant in the coding sequence of *FOXL2* in human adult-type granulosa cell tumors (AGCTs) (7). AGCTs are the most common sex-cord tumor type and occur most often after menopause (8). This somatic variant results in the p.Cys134Trp (C134W) substitution at the protein level affecting the sequence of the DBD and constitutes the pathognomonic feature of such tumors (9).

FOXL2 recognizes the consensus sequence 5′-RYMAAMA-3′ (R = G/A, Y = C/T, M = A/C), as most if not all of the members of the FOX family (3). More recently, chromatin immunoprecipitation followed by high-throughput sequencing (ChIP-seq) experiments in explanted primary granulosa cells have shown that FOXL2 peaks tend to map within the transcription units of its targets, in the introns and to intergenic regions (10,11) where it recognizes the consensus sequence 5′ RTAAAYA 3′ (10). Recent ChIP-seq experiments involving FOXL2 either wild-type (wtFOXL2) or C134W (mutFOXL2) expressed in the human immortalized granulosa cell line HGrC1 (12) showed that the sequence 5′ WMAACA 3′ (W = A/T) was highly enriched in both wt and mutFOXL2 peaks (matching the well-known consensus 5′ RYMAAMA 3′ (3)). However, they also found another highly enriched non-canonical composite motif for mutFOXL2 (i.e. 5′ AGHCAHAA 3′, H = non G), which seems to be a hybrid FHD-SMAD motif (12). According to this study, mutFOXL2 would acquire an ectopic capacity to bind SMAD4, forming mutFOXL2/SMAD4/SMAD2/3 complexes that recognize a large subset of unique DNA elements across the genome (13). In short, mutFOXL2 would hijack SMAD4 and thus drive the expression of genes involved in oncogenesis (12).

To identify partners of FOXL2 that might help it specifically recognize its targets, we have performed a proteomic analysis using co-immunoprecipitation in two cell lines naturally expressing FOXL2, followed by mass spectrometry. This approach allowed us to identify a core set of 255 (direct and indirect) partners common to both cell lines. A subsequent analysis showed, that in addition to TFs, FOXL2 co-precipitated with a series of complexes involved in RNA processing, chromatin remodeling and DNA replication and repair, pointing to unexpected interactions/roles of FOXL2 (14). These results confirm and extent our previous findings involving a yeast-two-hybrid screening showing interactions between FOXL2 and a series of TF but also with the sirtuin SIRT1 and the DNA repair protein XRCC6 (Ku70) (15). A more recent study has shown that after double-strand break induction, SIRT1 localizes to the nucleus and deacetylates FOXL2, thus releasing XRCC5 and XRCC6 from the complex with FOXL2, which leads to the formation of the Ku complex (a XRCC5/6 heterodimer). Accordingly, FOXL2 depletion enhances the recruitment of Ku to double-strand breaks, accelerating non-homologous end joining, inhibiting homologous recombination and leading to catastrophic genomic events (16). In addition, several works have shown that yeast forkhead TFs, Fkh1 and Fkh2, are global determinants of replication origin timing in a transcription-independent way and bind to a subset of origins to selectively recruit them to replication factories (17).

Building upon these unexpected protein-protein interactions and findings, we have set up to test the potential capacity of FOXL2 to interact with single-stranded nucleic acids. Using PCR-SELEX, we show that FOXL2 has the capacity to bind G_2_-rich RNA sequences. We also show that other FOX factors, namely FOXA1 and FOXO3a, are also able to interact with some of the preferred G_2_-rich RNA sequences bound by FOXL2. These results point to an unexpected feature of the FHD and paves the way for further research on the relevance and potential implications of these interactions *in vivo*.

## Materials and methods

### Plasmid construction

All enzymes are from New England Biolabs (Ipswich, MA), antibiotics from Sigma-Aldrich (St Louis, MO) and primers from Eurofins Genomics (Ebersberg, Germany). The Foxl2-1-376 (wt) and Foxl2-1-376-C134W coding sequences were subcloned into a pCDF-His-SUMO-DB backbone using the complementary single-strand annealing based method (18,19). Briefly, the coding sequences of Foxl2-1-376 and Foxl2-1-376-C134W were amplified from plasmids containing the full-length sequences of wtFOXL2 and mutFOXL2, respectively. After purification using a Gel/PCR extraction kit (NeoBiotech), each PCR product was mixed with pCDF-His-SUMO-DB plasmid digested by *Nde*I and T4 DNA polymerase was added to generate a complementary single-strand. The annealing reaction was then transformed in DH5a-T1R competent cells. Positive clones were validated by DNA sequencing.

### FOXL2 protein purification

Both FOXL2 proteins were expressed in and purified from *Escherichia coli* BL21 (DE3) pRARE2 cells. Briefly, bacterial cells were grown in 2 l of LB broth with 50 μg/ml spectinomycin and 34 μg/ml chloramphenicol at 37°C until *A*_600_ ∼1. Protein expression was induced with 0.5 mM IPTG followed by an incubation at 30°C for 4 h. Cells were lyzed by sonication in 20 mM Tris–HCl pH 8 @4°C, 500 mM NaCl, 1 mM DTT, 1 mg/ml lysozyme and 0.1% NP40, 1 mM AEBSF, Complete ULTRA Roche Protease inhibitor. Lysates were clarified by centrifugation at 150 000 × g and incubated with 20 mM imidazole on NiNTA resin (Qiagen) at 4°C for 4 h. The resin was washed extensively with buffer W1 (20 mM Tris–HCl pH 8 @4°C, 500 mM NaCl, 20 mM imidazole, 10% glycerol, 0.5% NP40, 1 mM AEBSF, 10mM Benzamidine, 2 μM pepstatine, 2 μM leupeptine), and then with buffer W2 (20 mM Tris–HCl pH 8 @4°C, 50 mM NaCl, 40 mM imidazole, 10% glycerol, 1 mM DTT, 1 mM AEBSF, 10 mM Benzamidine, 2 μM pepstatine, 2 μM leupeptine). The His-SUMO tag was cleaved by addition of His-SUMO-protease at 4°C overnight. The protein was eluted with buffer W2 containing 400 mM Imidazole and then directly loaded on a 1 ml Resource Q column (GE Healthcare) equilibrated with 20 mM Tris–HCl pH 8 at 4°C, 100 mM NaCl,1 mM DTT. A 30 ml gradient (100 mM-1 M NaCl) was applied. Fractions containing the purified proteins were pooled and the concentration was measured using Bradford reagent (Bio-Rad) with bovine serum albumin as a standard.

### PCR-selection amplification for a FOXL2 DNA/RNA-binding site

For double strand DNA (dsDNA) SELEX, a pool was synthesized using a DNA template containing a variable 26-base central region: ‘5 CAGATCAGTTCAGCGGATCGTGTCT- N_26_- GATGCGGAATTCAGTGCAACTGCAGC 3′. The template was converted into a dsDNA template using the primer pDSrev ('5 GCTGCAGTTGCACTGAATTCCGCATC 3′) and the Klenow polymerase fragment (New England Biolabs). The dsDNA products were purified on a 6% non-denaturing polyacrylamide gel (National Diagnostics) and retrieved by passive elution. The concentration of dsDNA was determined by UV spectroscopy at 260 nm (1 A260 unit = 50 mg/ml) as previously described (20).

For RNA SELEX, we prepared an RNA library of 91-mers, 5′ GGGAGAGUAUCCGUUGAGGCUGA-N_50_-AGAUCGGAAGAGCGUCGUGUAGG 3′, containing a variable 50-base central region, as previously described (20). Basically, a single-stranded DNA library ('5-CCTACACGACGCTCTTCCGATCT-N_50_-TCAGCCTCAACGGATACTCTCCC-3′) was first extended by Klenow polymerase (New England Biolabs) into a dsDNA template using the primer P72 ('5 TAATACGACTCACTATAGGGAGTATCCGTTGAGGCTGA 3′), which provides a promoter sequence for T7 RNA polymerase. The dsDNA products purified on a 6% non-denaturing polyacrylamide gel and retrieved by passive elution. Around 140 μg (approximately 2 nmol corresponding to 10^15^ template molecules) of dsDNA template was *in vitro*-transcribed at 37°C overnight in 2 ml of a reaction mix containing 40 mM HEPES (pH 7.5), 10 mM DTT, 12 mM MgCl_2_, 2 mM spermidine, 3 mM of each NTP (GE/Healthcare/Amersham Bioscences), 0.1% Triton X100, 1 u/ml inorganic pyrophosphatase (Roche) and home-made T7^Y639F^RNA polymerase. The RNA library was then concentrated using an YM30 Amicon Ultra filter-tube (Millipore) and purified on a 6% denaturing polyacrylamide gel electrophoresis. RNA concentrations were determined by UV spectroscopy at 260 nm.

Both dsDNA SELEX and RNA SELEX were performed using either recombinant wt or mutFOXL2. We performed six rounds of the following selection/amplification procedure. First, 0.75 mg of Dynabeads Protein A (Invitrogen/Dynal) were incubated 10 min at RT with a blend of our two anti-FOXL2 antibodies directed against the N-terminal and C-terminal peptides (21). The magnetic beads were then collected on magnets, washed two times with 200μl of washing buffer B (PBS, 0.02% Tween 20), resuspended with 200 μl of washing buffer B complemented with 10 pmol of FOXL2 (either wt or mutated) and incubated at RT, (agitation 600 rpm) for 10 min. The magnetic beads were then collected on magnets, washed two times with 200μl of washing buffer B, resuspended with 200 μl of Selection buffer S (10 mM HEPES pH7.5, 140 mM KCl, 25 mM NaCl, 1 mM MgCl_2_, 0.1 mM CaCl_2_ 0.001% IGEPAL) along with 1 nmol of library (either dsDNA or RNA) and incubated at 37°C (600 rpm) for 30 min. The magnetic beads were then collected, washed 3 times with 200 μl of buffer S, and the nucleic acids bound to FOXL2 were eluted in water at 85°C (600 rpm) for 10 min. Followed an ethanol precipitation. For RNA SELEX, the sequences were amplified by RT-PCR and *in vitro* transcribed as previously described (20). For dsDNA SELEX, the sequences were simply amplified by PCR. In the fourth, fifth and sixth rounds of SELEX, the library was first incubated with antibody-conjugated beads to remove RNA sequences that could bind to the Protein A-beads or to the antibodies.

### Deep sequencing and bioinformatic analysis

Aliquots of the library after each rounds of SELEX were prepared for deep sequencing on an Illumina system (Illumina, Little Chesterford, UK) as previously described (20). Basically, adaptor and indexing sequences required for Illumina multiplexing sequencing were added to the DNA libraries by PCR. The PCR products were purified on a 3% agarose gel and purified using a Monarch® Gel extraction Kit (New England Biolabs) following manufacturer's instructions. Samples were then mixed and loaded with 10% PhiX into a flow-cell, sequenced and de-multiplexed according to the Illumina's instructions. All FASTQ files were processed using several home-made scripts that were used sequentially to analyze the results and generate the corresponding graphs (Excel and GraphPad Prism). The sequencing analysis workflow and relevant statistics for DNA SELEX are displayed in Supplementary Figures S1 and S2. In short, sequences corresponding to the variable region between the primer sequences were recovered. Then, sequences that contained at least one base with a quality score (*Q*) below 30 were removed before being saved in a FASTA format. This quality score can be converted to a probability of error (*P*) using the formula *P*  =  10^(–^*^Q^*^/10)^. Thus, the recovered sequences contain bases with a potential probability of error below 0.001 (1 in 1000). The frequency of each sequence in the library was calculated. All sequences were then compared to each other using the Levenshtein distance in order to cluster them into families (22). Enrichment of sequence motifs were searched using MEME (Multiple Em for Motif Elicitation) (23). The sequencing analysis workflow and relevant statistics for RNA SELEX are displayed in Supplementary Figures S5 and S6. Information on the pipeline and access to it can be requested at aptamer-MIRCen@cea.fr.

### Immunoprecipitation and competition experiments

To test the specificity of the interaction between wtFOXL2 and potential G4-forming RNAs or the corresponding ssDNA counterparts, we compared the frequency of retention of ssRNA or ssDNA aptamers from an equimolar amount of four sequences (RF000: 5′ GAGGUAUUAAGAGGCGGAGGCGGAAGGAUGGCGGAGGACCGACAGAGAAG 3′, RF001: 5′ CGUCAUUUGGUGGAUGGCGGAAGGAGGCGGAGGACGGGAAAUUCUGGAAA 3′, RF002: 5′ UCGGACUUUGGUGGUUGGAGGACGGAUGGACGGAGGUUUAUUAGGCUCGC 3′, RF004: 5′ AUCUGCGACUGGCUGGAGGUGGACGGAUGGCUGGAGGCGAAAUUGGCUUA 3′) and one ‘control’ sequence unable to fold into G4 (scr50: 5′ GGGACACUCGCUGUUUUCGAAAUUACCCUUUAUGCGCGGGUAUUGAACCA 3′) (10mM each). Antibodies (Ab) against FOXL2 were bound to Dynabeads® Protein A in 0.02% PBS-Tween buffer. Then, 100 pmol of purified protein (FOXL2 wt or C134W) were incubated with the bead-Ab mixture. After 30 min, the beads were rinsed twice in the selection buffer 1× (HEPES 10 mM, KCl 140 mM, NaCl 25 mM, MgCl_2_ 1 mM, CaCl_2_ 0.1 mM, Igpal 0.001%) before being put in the presence of an equimolar mixture of aptamers (50 nM total) and 100nM of competitor tRNA from yeast (R5636 Sigma) for 30 min at 37°C under agitation (600 rpm). A control without FOXL2 protein (i.e. dynabeads + antibody) was also included in the experiment. The mixture of aptamers and competitor was freshly prepared and incubated for 5 min at 85°C, then 3 min in ice and 5 min at 37°C and added immediately to beads-Ab-protein complex. The beads were rinsed three times in selection buffer before aptamers elution in water (10 min at 85°C, 600 rpm). Aptamers were then precipitated and in the case of RNA, reverse transcribed, before sequencing. For competition experiments in the presence of LiCl, only the composition of the buffer was changed: HEPES 10 mM, LiCl 140 mM, MgCl_2_ 1 mM, CaCl_2_ 0.1 mM, Igpal 0.001%. A control without FOXL2 protein (i.e. dynabeads + antibody) was also included. Specific details of other similar competition experiments are given in the Results section and figure legends.

### *FOXL2* knock-down (KD) experiment in KGN cells

Crispr-edited KGN cells harboring two copies of wild-type *FOXL2* were transiently transfected in six-well plates (same conditions as above) with 30 pmol of a pool of three different siRNAs against FOXL2 for RNA-seq experiments (Dharmacon, ON-TARGETplus siRNAs, pool KD1: J-009075-06-005, J-009075–07-005, J-009075-08-005, KD2: J-009075-06-005, J-009075–07-005, J-009075–09-005 and KD3: J-009075–06-005, J-009075-08-005, J-009075-09-) or an equal amount of the control/scrambled siRNA (Dharmacon, D-001810-10-05) using Lipofectamine RNAiMax (Thermo Fisher Scientific). Six hours after transfection, the mix was replaced by culture medium and cells were allowed to grow for 24 h before performing subsequent experiments.

### RNA extraction and sequencing

For the KD experiment, RNA extraction was performed 30 h after transfection, using the TRI-reagent, according to the supplier's instructions (MRC). The KD efficiencies were evaluated by RT-qPCR. cDNA synthesis was performed with 1 μg of total RNAs after DNase treatment (SuperScript IV RT enzyme according to the manufacturer's protocol (Thermo Fisher Scientific)). RT-qPCR was performed using the GoTaq qPCR Master Mix 5X (Promega, Madison, Wisconsin) in the Stratagene Mx3000P qPCR System. 1 μg of total RNAs underwent high throughput sequencing at Genom’IC, Institut Cochin, Paris, France). Specifically, total RNA quality was assessed on an Agilent Bioanalyzer 2100, using RNA 6000 pico kit (Agilent Technologies). Directional RNA-Seq Libraries were constructed using the TruSeq mRNA Stranded library prep kit (Illumina), following the manufacturer's instructions. Final library quality was assessed on an Agilent Bioanalyzer 2100, using an Agilent High Sensitivity DNA Kit. Libraries were pooled in equimolar proportions and sequenced on Single Read 75 pb runs, on an Illumina NextSeq500 instrument, using NextSeq 500 High Output 75 cycles kits. Fastq files were then aligned using STAR algorithm (version 2.7.6a). Quality control of the alignment was performed with Picard tools (version 2.8.1) and reads were then counted using RSEM (v1.3.1). The statistical analyses on the read counts were performed with R (version 3.6.3) and the DESeq2 package (DESeq2_1.26.0). The *P* values were adjusted for multiple testing using the Benjamini and Hochberg correction, and those with an adjusted *P* value < 0.05 were considered to be significant. Data were deposited in the Gene Expression Omnibus (GSE227040).

## Results

### FOXL2 recognizes G_2_-rich RNAs *in vitro*

**Figure 1.**
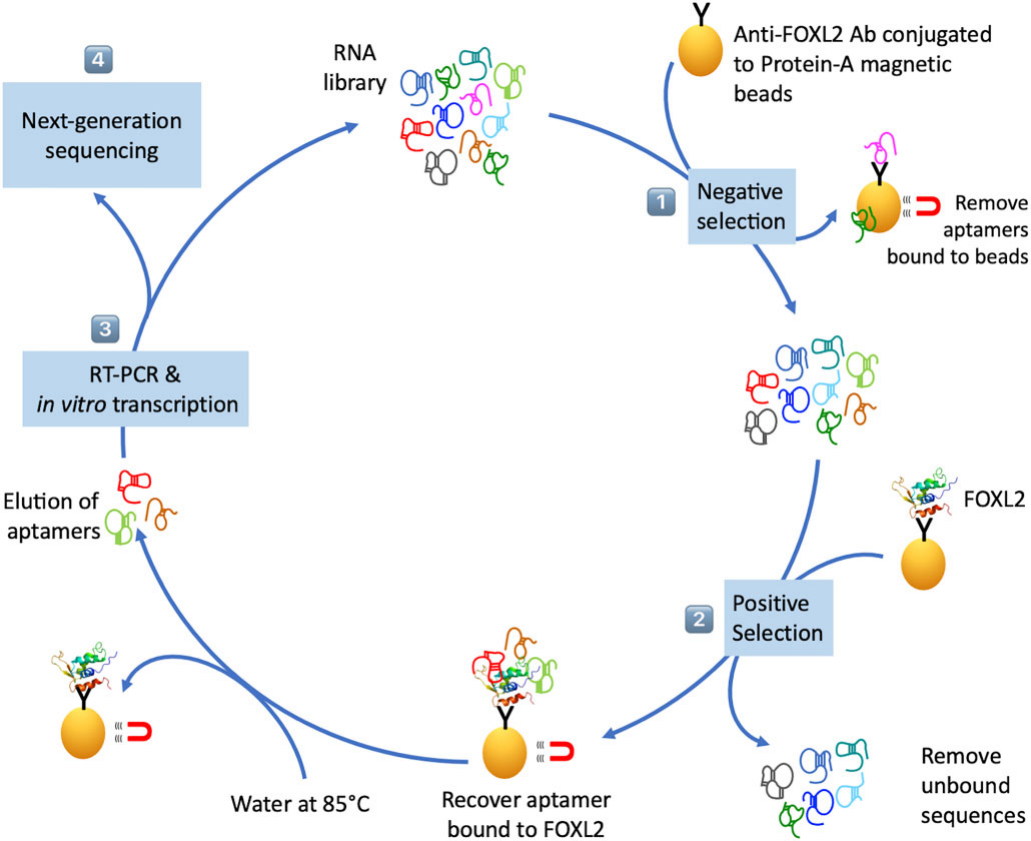
Schematic representation of *in vitro* selection against FOXL2. A nucleic acid library (dsDNA or ssRNA) is first incubated with anti-FOXL2 antibodies (Ab) previously fixed on Protein A magnetic beads. Oligonucleotides that pass this negative selection are then incubated with FOXL2 immobilized on anti-FoxL2 antibodies. After several washes, bound aptamers are eluted by denaturation in water at 85°C before being amplified by RT-PCR and *in vitro* transcription for ssRNA, or only PCR for dsDNA. At each selection round, an aliquot of the library is retrieved for HTS.

**Figure 2.**
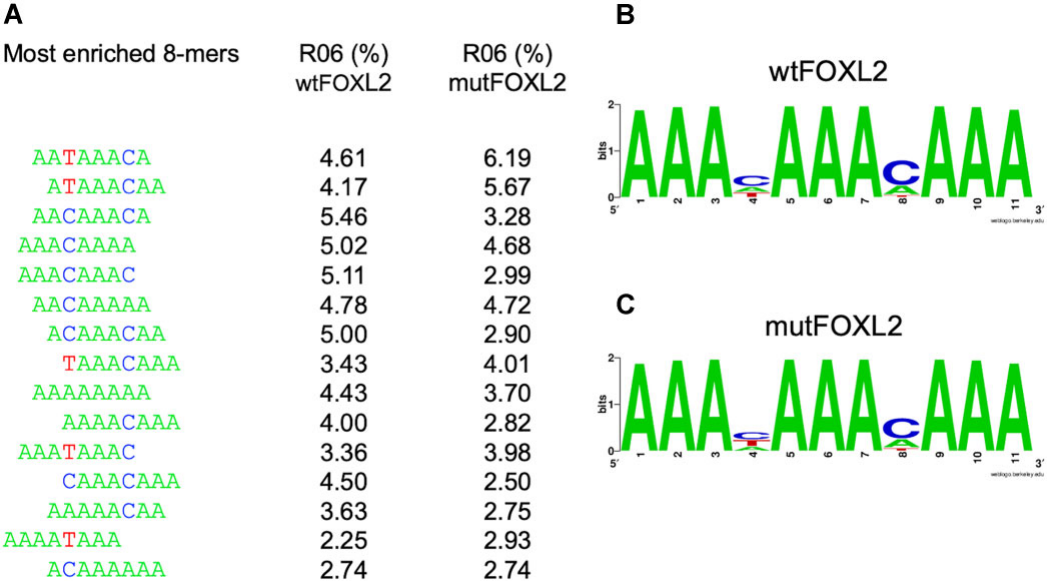
Topmost enriched 8-mers identified by dsDNA SELEX for wtFOXL2 and mutFOXL2. (**A**) Alignment of the most enriched 8-mers after six rounds of selection. (**B**) Logo calculated using the frequency of each k-mer in the last round of SELEX using wtFOXL2. (**C**) Same as B but for the SELEX using mutFOXL2.

Here, we have used purified bacterially-expressed FOXL2 proteins (wt and mutFOXL2) in order to explore whether they are also capable of binding single-stranded nucleic acids. First of all, to show that both recombinant proteins were active, we performed a classical dsDNA SELEX. The general principle of the Selex experiment is illustrated in Figure 1. Sequences bound by each FOXL2 version were isolated by immunoprecipitation using our anti-FOXL2 antibodies (21). Next, we analyzed the retained DNA fragments through six rounds of selection by high-throughput sequencing (HTS). After some filtering steps, we focused on sequences that could be detected in at least one round with a frequency >0.003% (the sequencing analysis pipeline and the relevant statistics can be found in Supplementary Figures S1 and S2). The evolution of the global nucleotide composition during SELEX for wt and mutFOXL2 are displayed in Supplementary Figures S3 and S4 respectively. Figure 2A shows an alignment of the most enriched 8-mers found after 6 rounds of selection using either wt or mutFOXL2. The sequence logos in Figure 2B and C were computed using the frequencies of each k-mer in the last round of selection. They do not show any obvious difference between the sequences recognized by wt and mutFOXL2. The high affinity binding site corresponds to 5′ AAAHAAA 3′ (H = non G), a sequence in agreement with the general consensus for the FOX family of TFs (3,10,12).

**Figure 3.**
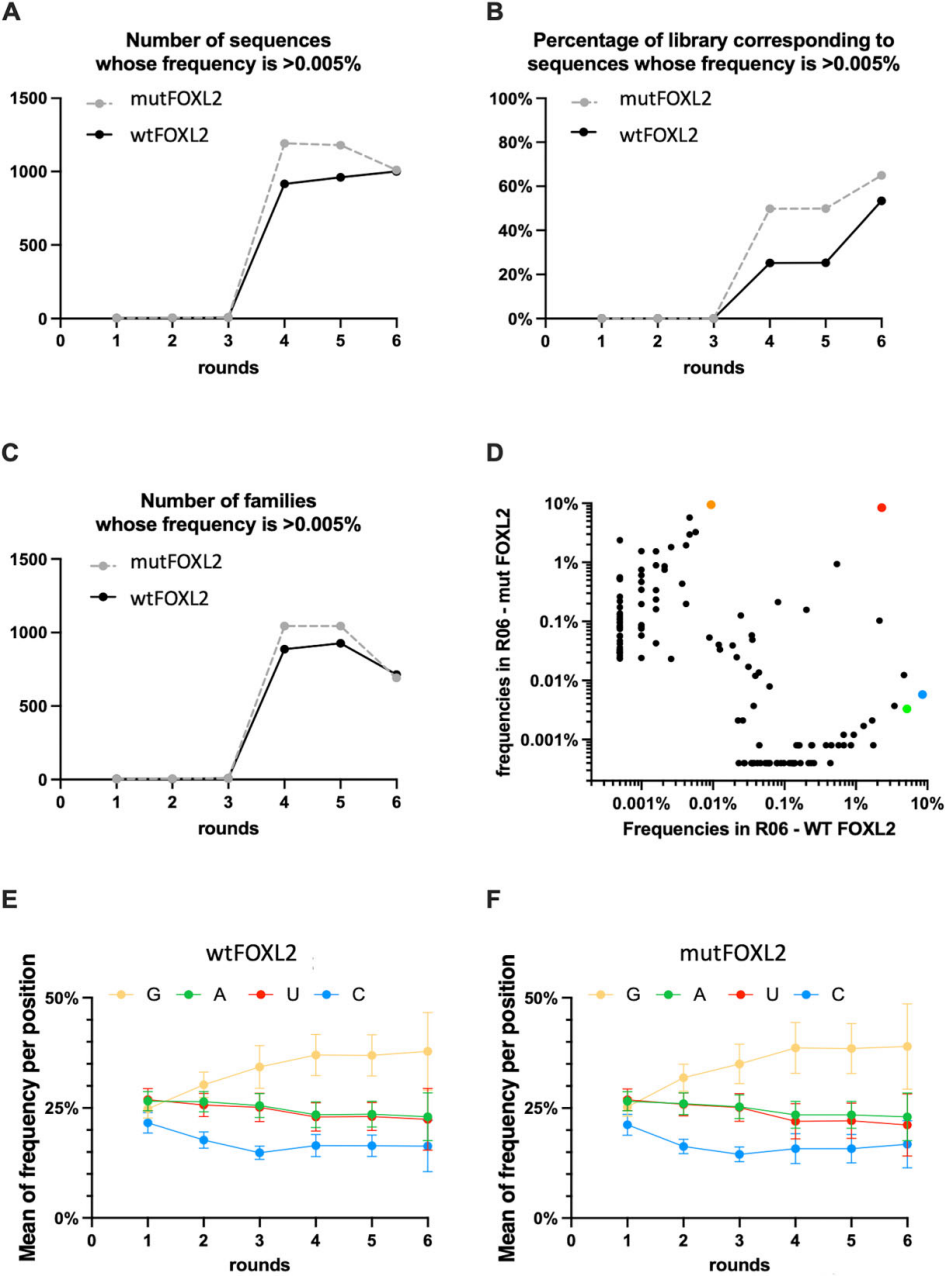
Deep sequencing analysis of RNA SELEX. The libraries after several rounds of SELEX against wtFOXL2 or mutFOXL2 were analyzed by HTS. (**A**) Number of sequences with a frequency higher than 0.005% in at least one round. (**B**) Percentages of sequences with a frequency higher than 0.005% in at least one round. Those sequences were clustered into families based on an edit distance of 10. (**C**) Evolution of the number of families along the SELEX process. (**D**) Comparison of the frequencies of the topmost enriched families in the last round of both SELEX experiments for wt versus mutFOXL2. The colored dots correspond to the four families, which were selected for binding evaluation in competition experiments (orange, blue, red and green stand for RF000, RF001, RF002 and RF004, respectively). (**E**) Evolution of the average nucleotide frequency per position for wtFOXL2 and (**F**) for mutFOXL2. Note that the starting point (round 0) is 25% (random composition, no bias) and the enrichment in G from one round to the next.

To further analyze the ability of FOXL2 to bind nucleic acids, we tested whether the wt and mutFOXL2 proteins could interact with ssRNA or not. For this, we performed an RNA-SELEX experiment. As for dsDNA, we performed six rounds of selection. After HTS, we monitored the quantitative evolution of sequences, focusing on those that could be detected in at least one round at a frequency >0.005% (i.e. 50 copies per million sequences). The sequencing analysis pipeline and relevant statistics for RNA SELEX can be found in Supplementary Figures S5 and S6. The evolution of the global nucleotide composition for wt and mutFOXL2 are displayed in Supplementary Figures S7 and S8. All the sequences were clustered into families. Every family was composed of similar sequences with no more than 10 substitutions, insertions or deletions (i.e. a Levenshtein distance of 10). The sequences of most families were separated by a maximum Levenshtein distance of 5. As shown in Figure 3A and B, we detected a few sequences with a frequency >0.005% in the library until round 3. They collectively represented less than 0.1% of the total population. However, their number increased up to about 1000–1200 sequences (for wt and mutFOXL2 respectively) from round 3 to round 5. Their frequency in the pool rapidly increased, to finally represent >50% at round 6 (Figure 3B). The fact that the number of families with a frequency > 0.005% decreased after round 5 (Figure 3C) demonstrates a preferential amplification of a few related families. Figure 3D displays a comparison of the frequencies of the most enriched families in the sixth and last round of selection for wt and mutFOXL2. Four representative sequences of four families (RF000, RF001, RF002 and RF004) were chosen for further studies. Figure 3E and F shows that the base frequency for all sequences showed a consistent enrichment in G from one round to another (from 25% up to about 40%). Twenty-five percent being the random expectation for a non-biased library (see also Supplementary Figures 1 and 2), the observed enrichment suggests that both FOXL2 versions prefer G-rich RNA sequences, which is not the case when they interact with dsDNA, characterized by an avoidance of G (Figure 2 and Supplementary Figures S3 and S4).

**Figure 4.**
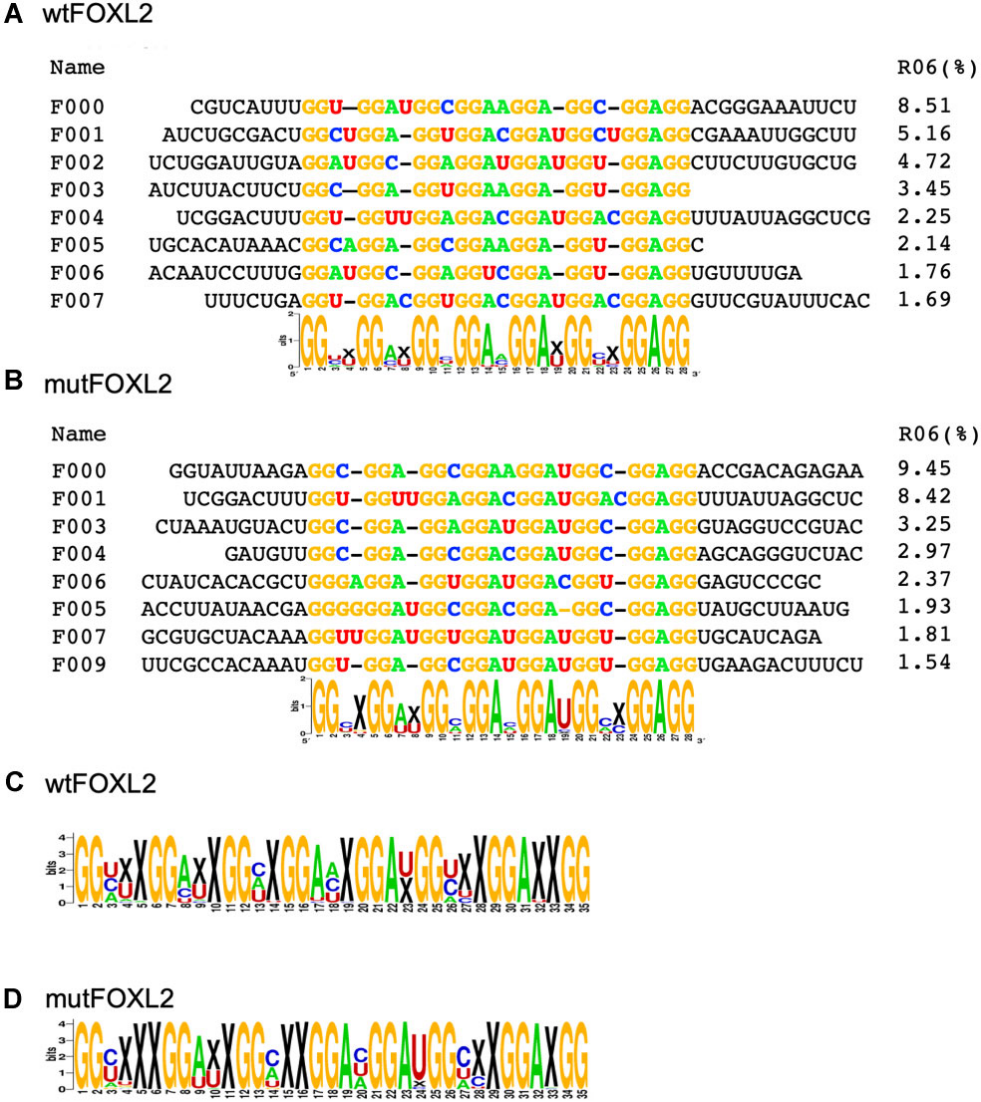
G_2_-rich RNA motifs identified by deep sequencing. (**A**) Alignment of the eight most enriched families that contain a conserved motif with eight G doublets (in bold) and their frequency after six rounds of selection using wtFOXL2. The logo at the bottom was calculated based on the abundance of each family in the last round of SELEX. (**B**) Same as in Figure A for mutFOXL2. (**C** and **D**) Logos computed by considering the abundance in the last round of the 100 most enriched families in SELEX experiments using wt or mutFOXL2, respectively.

Multiple alignments of RNA families enriched during both SELEX experiments (i.e. using wt and mutFOXL2) revealed that most of them contain a G_2_-rich sequence (Supplementary Tables S1 and S2, respectively). The 8 most enriched families containing a G_2_-rich sequence represented approximately 30% of all sequences from round 6 for each protein version. These families were used to establish consensus sequences (Figures 4A and B). This allowed us to uncover a motif containing 8 G doublets, the presence of which was obvious for both wt and mutFOXL2, with only minor differences in the proportions of the intervening nucleotides or gaps depending on the protein version. More relaxed versions of the consensus sequences were obtained by computing the Logos using the 100 most enriched families (Figure 4C and D). We observed differences between the logos computed over the eight most enriched families and those computed over 100 families, and also between the logos for wt and mutFOXL2 when the computation was performed over 100 families.

**Figure 5.**
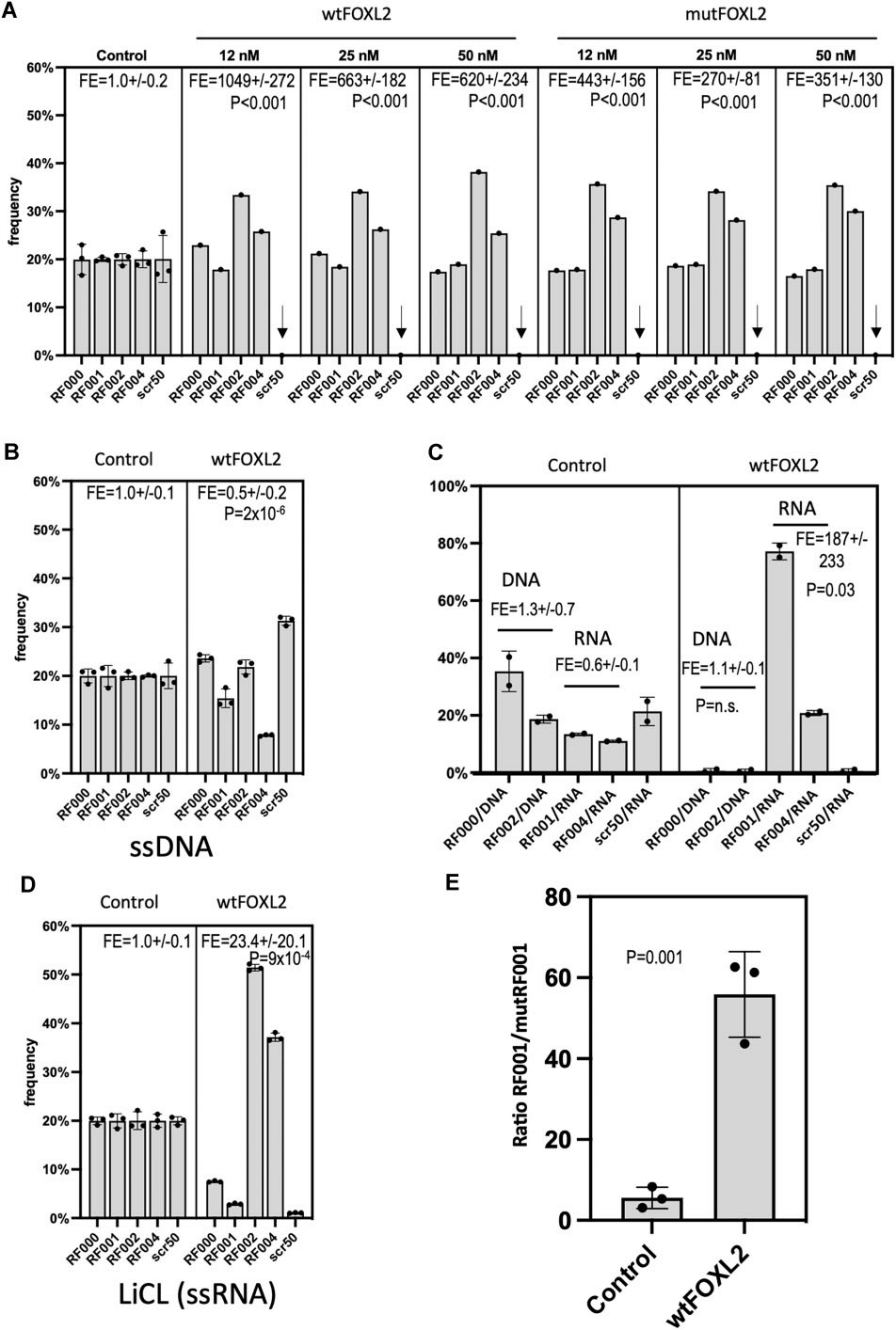
Specificity of the interactions between FOXL2 and G_2_-rich RNAs. (**A**) Relative retention frequencies of four G_2_-rich RNA aptamers (RF000 to RF004) and a control one (scr) mixed in equimolar amounts by different amounts of immobilized wt and mutFOXL2 proteins. The G_2_-rich aptamers were retained in much higher proportions than the control sequence which was basically absent (vertical arrows). The leftmost panel (here and in B and C) refers to a control condition without FOXL2 protein but in the presence of Dynabeads + Antibody (recovered sequences = non-specifically bound). To statistically assess this apparent difference, we calculated a fold enrichment (FE) as the ratio of retained RF00x/scr50 sequences for each replicate and then applied a two-tailed Mann–Whithney *U* test for comparing the means over all the computed ratios in the absence (control condition) or presence of FOXL2. The FE and associated *P*-values are displayed. (**B**) A similar experiment explored whether wtFOXL2 interacted with the same sequences in DNA chemistry. The five sequences were retained in rather similar proportions suggesting that the recovered DNA is likely to be non-specifically bound. When several replicates were available, a two-tailed Student's *t*-test was used to calculate the *P*-values (here and throughout this paper). (**C**) Competition experiment similar to those described above performed with sequences RF000 and RF002 in the form of DNA and RF001 and RF004 in the form of RNA, in the presence of our control sequence scr50. The FE of the DNA or RNA sequences with respect to our control (scr50 in RNA) are displayed. The significance of the differences in FE between the control and the wt FOXL2 conditions was estimated with a two-tailed Mann–Whitney *U* test. (**D**) Competition experiment similar to those described above performed in the presence of LiCl (G4 destabilizing). Retention of RF000 and RF001 drastically decreased (more likely to form G4). Sequences RF002 and RF004 displayed enrichments clearly higher than that of the control (less sensitive to Li+). (**E**) Competition experiment performed with the sequence RF001 (5′CGUCAUUUGGUGGAUGGCGGAAGGAGGCGGAGGACGGGAAAUUCUGGAAA 3′) along with its mutated version, mutRF001, predicted not to form rG4, where all G doublets were disrupted (while keeping the same base composition. mutRF001: 5′ CGUCAUUUGAGUGAGUGAGCGAGAGAGAGUGCGUGAGAGACGAGAGCGUG3’). The ratio RF001/mutRF001 (i.e. RF001/mutRF001) was more than 10 times stronger in the presence of FOXL2 than in the control condition.

**Figure 6.**
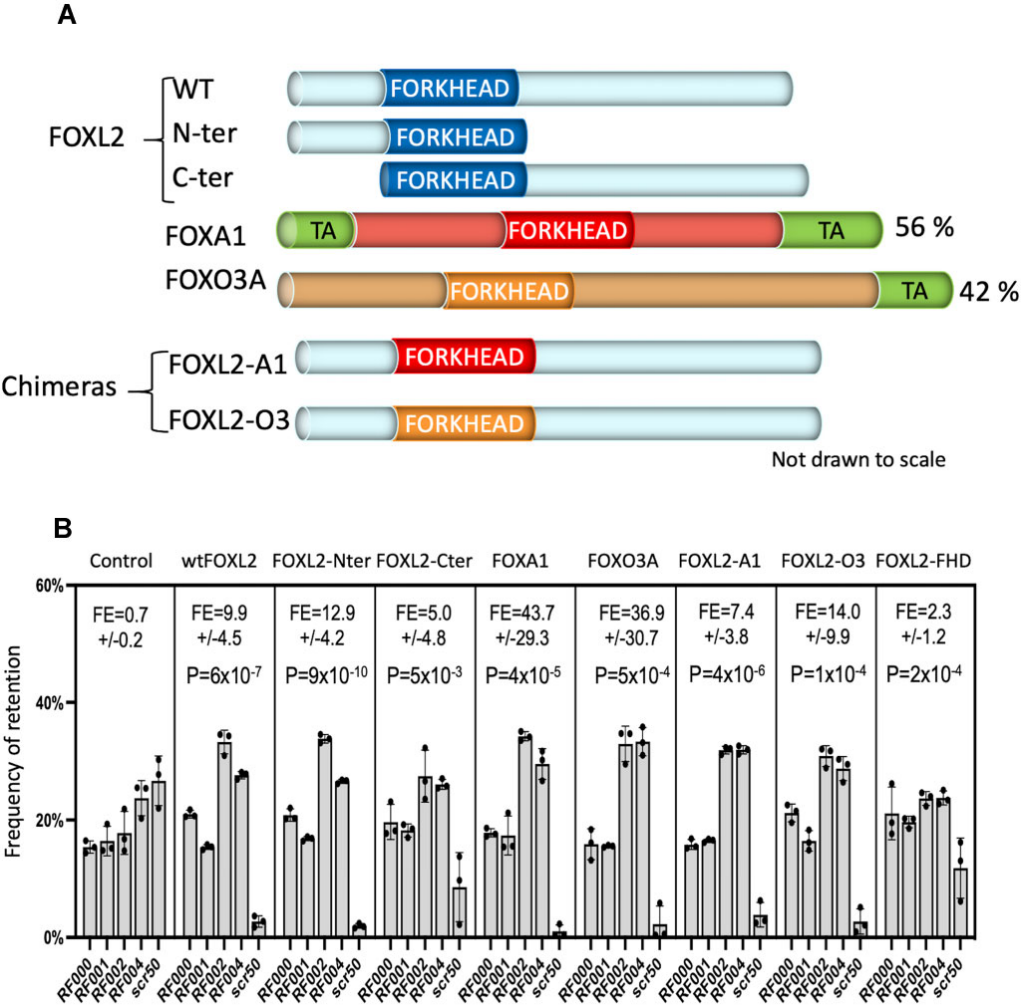
The FHD is a potential ssRNA-binding domain. (**A**) Schematic representation of the FHD-containing proteins used in the experiment. (**B**) The graphs represent the relative retention frequencies of four RNA aptamers (RF000 to RF004) and a control one (scr50) in equimolar amounts by wtFOXL2, FOXL2 without its N-ter, without its C-ter but keeping an intact FHD, FOXA1, FOXO3A, chimeric FOXL2 with the FHD of FOXA1 and chimeric FOXL2 with the FHD of FOXO3A and by the FHD alone. The FE and associated p-values (in a comparison with the control—no FOX protein—condition) are displayed.

In order to obtain a further proof of the specificity of the interaction between wt FOXL2 and G_2_-rich RNAs, we compared the relative retention frequencies of four G_2_-rich (RF000 to RF004) and a control one (scr50). For this, increasing amounts of FOXL2 proteins immobilized on magnetic beads were incubated with a mix containing equimolar amounts of the four RF and the control (scr50) sequences. After washing and eluting the fixed RNA-aptamers, deep sequencing of the cDNAs showed that the control sequence was found in a much lower proportion than each of the G_2_-rich aptamers (Figure 5A). To statistically assess this apparent difference, we calculated a Fold Enrichment (FE) as the ratio of retained RF00x/scr50 sequences (*x* = 0–4) for each replicate and then compared the means computed over all of the ratios in the absence or presence of the relevant protein (e.g. FOXL2). This showed that existence of statistically significant differences for each protein concentration. Although only one replicate for each protein concentration was available, the consistency of the results across the various concentrations shows the specificity of the interactions between FOXL2 and the RF sequences. This is further supported by the results displayed in Figure 6.

Using a similar approach, we explored whether wt FOXL2 was able to interact with the same sequences (i.e. RF000 to RF004) in DNA chemistry. HTS revealed no clear preference for the RF sequences (Figure 5B), which is expected for DNA sequences non-specifically bound to the components of the reaction mixture. Instead, we detected an FE < 1, driven by a slightly greater retention of the scr50 sequence. This suggests that FOXL2 specifically interacts with an RNA structure formed by G_2_-rich sequences and not with the same sequences in the form of ssDNA. To provide further evidence of this, we performed a competition experiment in which sequences RF000 and RF002 in the form of DNA were mixed with RF001 and RF004 in the form of RNA molecules, in the presence of our control sequence scr50 in RNA chemistry. Figure 5C shows a strong and significant enrichment of RF001 and RF004 showing beyond any reasonable doubt the preference of FOXL2 for G_2_-rich RNA over DNA or scr50.

The existence of G doublets separated by one to three nucleotides is compatible with the formation of G-quartets (rG4) in the RNA sequence in our experimental conditions. A general consensus sequence would be G_2_N_1–4_G_2_N_1–3_G_2_N_1–3_G_2_AN_1–2_G_2_AU_0–1_G_2_N_1–3_G_2_AN_0–2_G_2_. We used QGRS Mapper to determine the probability of G-quadruplex formation by this G_2_-rich motif (24). As expected, the G-Score for 5′ GGNNGGNNGGNGGANGGANGGNNGGAGG 3′ was high (i.e. 21). Similarly, high scores were obtained for the more relaxed sequences with longer spacings between the G doublets. To gather evidence that the ssRNA aptamers can adopt a G4 structure, we performed competition experiments similar to those described above in the presence of LiCl (instead of NaCl or KCl), which is supposed to be unfavorable to the formation of quadruplexes ((25) and references therein). If all of the potentially rG4-forming sequences were equally sensitive to Li, we would expect similar retention of the control sequence and of RF sequences. The retention of RF000 and RF001 drastically decreased, as expected, suggesting that such sequences can adopt an rG4 structure recognized by FOXL2. However, sequences RF002 and RF004 still displayed enrichments clearly higher than that of the control and of RF000 and RF001, showing that they are less sensitive to the effect of Lithium. Potential structural differences between RF002 and RF004 versus RF000 and RF001 require investigation because the former were more enriched in the presence of Na^+^/K^+^ and remained more enriched in the presence of Li^+^. However, note that the retention frequencies displayed in Figure 5 are relative and not absolute quantities. Since the percentages add to 100, a decreased retention of scr50, RF000 and 001 mechanically translates into an increase of RF002 and 004 but this does not necessarily imply that they are more efficiently retained (i.e. in absolute terms, compared to a condition in the presence of Na and/or K). Finally, we performed a competition experiment involving RF001 and a mutated version (mutRF001) where all G doublets were disrupted while keeping the same base composition. As expected, mutRF001 was predicted not to form rG4. Figure 5E clearly shows a strong enrichment of RF001 over mutRF001.

### The forkhead domain mediates the recognition of G_2_-rich RNAs

As the FHD is the only domain of FOXL2 predicted to have a stable structure, we hypothesized that it could also be responsible for RNA-binding. To test this idea, we compared the frequencies of retention of RF and control sequences in the same conditions as above, by (i) FOXL2 without its N-ter but keeping an intact FHD, (ii) without its C-ter but keeping an intact FHD, (iii) FOXA1, (iv) FOXO3A, (v) chimeric FOXL2 with the FHD of FOXA1, (vi) FOXL2 with the FHD of FOXO3A and (vii) FOXL2’s FHD alone. All proteins were tagged with a V5 epitope, were expressed in HeLa cells and immunoprecipitated with an anti-V5 antibody for competition experiments (RF001-004 plus scr50, as described above). HTS of the cDNAs of the retained RNA aptamers showed that the control sequence was always found in a much lower proportion than the G_2_-rich RNA sequences (Figure 6A, B) suggesting that the only common domain among those proteins, namely the FHD, is indeed the G_2_-rich RNA-binding domain. This was confirmed by the significant enrichment found in the experiment involving the FHD of FOXL2 alone (rightmost panel of Figure 6B).

### Genes responding to *FOXL2/foxl2* knock-down display a non-random presence of G_2_-rich sequences.

**Figure 7.**
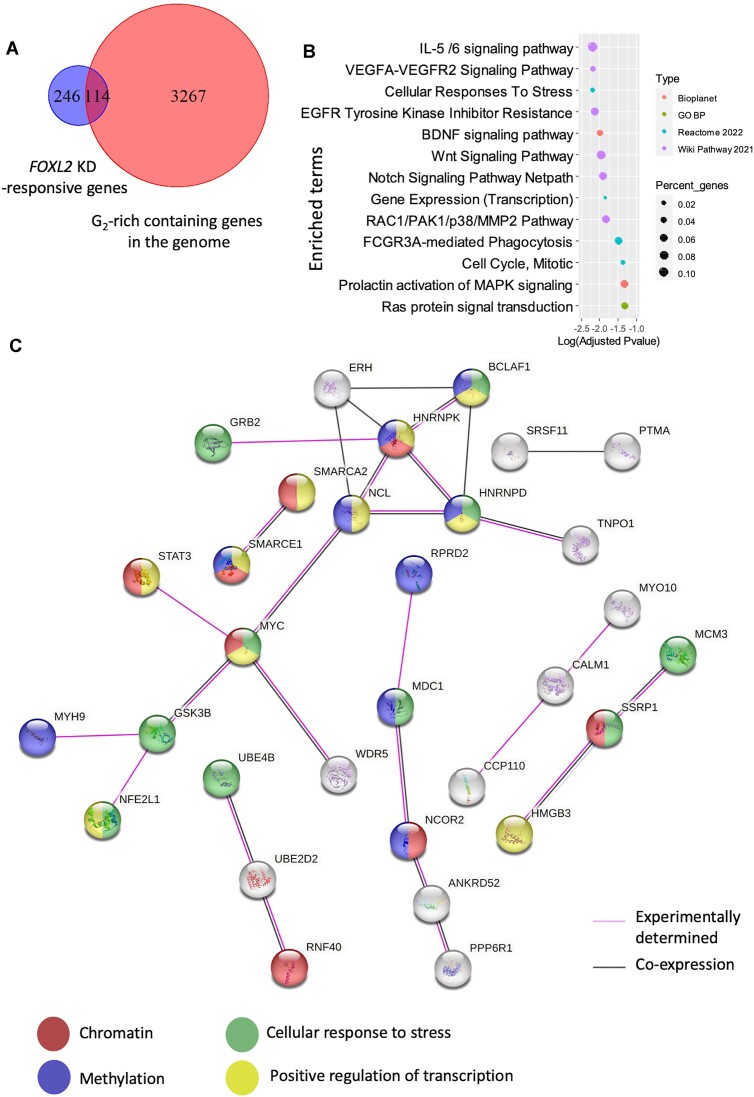
The genes deregulated by a FOXL2 knock-down are enriched in G2-rich sequences. (**A**) Overlap between the 360 Differentially Expressed Genes (DEGs with adjusted *P*-values < 0.05) responding to FOXL2 KD and genes/transcripts containing *in vitro* rG4 in both HeLa and HEK293T (27). (**B**) Bubble plot of an ORA with the 114 DEGs found in the overlap displayed in (A). If a GO term occurred several times in different databases only the one with the smallest adjusted *P*-value was kept. (**C**) DEGs mapping to the overlap are involved in densely connected (sub)networks of protein-protein interactions (produced using String (38) (https://string-db.org) with default parameters based on experimental interactions or co-expression).

In an attempt to explore a potential functional impact of FOXL2-ssRNA interactions, we knocked-down (KD) *FOXL2* in genome-edited human KGN cells bearing two wt alleles of *FOXL2*, to assess whether a significant fraction of Differentially Expressed Genes (DEGs) bore G_2_-rich sequences or not. According to our RNAseq data, *FOXL2* experienced a 3.6-fold reduction of expression (i.e. only 27% of FOXL2 expression remained after the KD, Supplementary Table S3). This result was corroborated by RT-qPCR (25% of remaining FOXL2 expression). This experiment revealed 360 DEGs (with adjusted *P*-values < 0.05). Because computational studies have shown that mRNA can harbor potential G4s (25–27), we determined the overlap between the list of DEGs and a list of transcripts supposed to form rG4s, identified through a high throughput analysis of *in vitro* stops to reverse transcription (RT stops). The comparison of the list of 360 DEGs and with that of 3381 genes containing rG4s in both HEK293T and HeLa cells according to (27) pointed to 114 DEGs potentially bearing rG4s (*P* < 3.1e^−12^, *P*-value calculated using http://nemates.org/MA/progs/overlap_stats.html, Figure 7A). An over-representation analysis (ORA) using Enrichr (28) of these 114 DEGs showed a series of non-random signals. Indeed, various signaling pathways (FGF, MAPK, VEGFA-VEGFR2, PI3K, etc.) and cellular processes (RNA pol II-mediated transcription, methylation, cell cycle/mitosis, etc.) were statistically enriched (Figure 7B). In addition, thirteen of the 114 gene products were involved in a densely connected network of protein-protein interactions, as shown in Figure 7C.

We extended this analysis to data from a previous study from our group in which we depleted *Foxl2* (KD) for 30h in mouse primary granulosa cells (11). In this experiment, *Foxl2* displayed a fold reduction of 2.92 (Supplementary table S3). We focused on 1364 statistically significant DEGs (*P*< 0.05 after a Benjamini–Hochberg correction) having, in addition, a Fold Change ≥1.4 in either direction (activation or repression, Supplementary table S4). Next, we compared the list of DEGs with a list of 773 genes harboring 1 141 regions displaying strong RT stop signals (10-fold above background, found in mESC) that were the best candidates to display rG4 folding, according to (27). An ORA of the 38 DEGs found in the overlap (*P* < 0.016) showed again non-random signals. These DEGs were involved in pathways and functions such as TGF-beta, HIF1, VEGFA, adherens junctions, apoptosis, etc. (Supplementary table S4).

## Discussion

As shown above, the dsDNA sequences recognized by both wt and mutFOXL2 contain the core 5′ AAACA 3′ (and variants), previously described for FOXL2 (3,10,12). This result suggests that the C134W mutation does not alter the intrinsic DNA sequence binding specificity of FOXL2. This result lends credence to the proposal that a perturbation of the interaction of mutFOXL2 with other partners such as SMAD3/4 underlies cancer (12,13,29). In short, wt and mutFOXL2 have an intrinsic capacity to recognize the same DNA consensus sequence. However, in the context of granulosa cells, mutFOXL2 would form ectopic complexes with SMAD4 (and probably with SMAD2/3) that recognize novel target genes able to trigger an oncogenic program (12,13)

Our most interesting finding is that FOXL2, a canonical TF, can specifically bind RNA aptamers with G_2_-rich RNA sequences out of a random population. The affinity for the same sequences in ssDNA chemistry seems to be much lower or inexistent, as shown in Figure 5B, C. Moreover, we suggest that the FHD is itself the RNA-binding domain. It is, indeed, the only protein domain common to FOXL2, FOXA1 and FOXO3a. The results of the competition experiments displayed in Figure 6 show Fold Enrichments (FE) lower than those reported in Figure 5A (i.e. for purified proteins). This is probably due to the lower FOX protein input in the former experiments (i.e. with immunoprecipitated proteins), which increases the impact of non-specific interactions between ssRNA and the components of the reaction mixture on the results. This was particularly obvious in the competition experiment with the FHD of FOXL2 alone. In our hands, the FHD is less well expressed than the entire FOXL2 protein, which explains why in the competition experiment the FE although significant was less drastic. The basic isoelectric point (p*I*) of FOXL2 or, more specifically of the FHD (ranging from 9 to 10 for a variety of paralogs, see ref.(30)) can facilitate the interaction with the aptamers but cannot explain its specificity. Interestingly, although differences between the logos for wt and mutFOXL2 in RNA SELEX were minor when focusing on a restricted number of sequence families, we did observe differences between the two protein versions when logos were computed over 100 families. The biochemical and biological significance of this finding (if any) remains to be studied.

The G_2_-rich sequences bound by FOXL2 and the FHDs tested contained 8 G doublets. Such G_2_-rich sequences can form structures based on the non-canonical Hoogsteen-type base pairing, leading to a coplanar arrangement of four Gs (quartet). The stacking of at least two quartets, stabilized by a monovalent cation (Na + or K+) constitutes the G4 (31). The competition experiments in the presence of the rG4 destabilizing cation Li + and between R001 and mutR001 (predicted to be unable to form rG4s) suggests that the RNA aptamers studied here can potentially form rG4s.

G-quadruplex structures can be predicted by computational means (32) and can be identified in the genome by ChIPseq using specific anti-G4 antibodies (31). *In vitro* and *in vivo* experiments have shown that such G4 RNAs play important roles in RNA metabolism, pre-mRNA processing (including splicing and polyadenylation) and translation (26). Interestingly, we found that a statistically significant fraction of potential direct and indirect FOXL2 targets (i.e. DEGs in the *FOXL2/Foxl2* KD experiments using a human cell line and mouse primary granulosa cells) can form rG4s at least *in vitro* (27). Moreover, such DEGs bear non-random ORA signatures that deserve exploration. FOXL2 being able to (directly or indirectly) activate or represses transcription (10,11), the fact the we do not find a consistent effect (only activation or repression) on the expression levels of the potential rG4-containing FOXL2 (direct and indirect) targets is not surprising. Although interesting, this preliminary analysis has several limitations. First of all, it provides only indirect functional evidence focused on potential FOXL2 transcriptional targets. This excludes *de facto* effects on other transcripts. Moreover, our main analysis focused on transcripts bearing rG4s that are common to KGN, HeLa and HEK293T cells, meaning that a part of the KGN transcriptome was not screened (i.e. rG4-containing FOXL2 targets not expressed in HeLa and HEK293T cells). Finally, Guo and Bartel have shown that thousands of mammalian mRNA regions that can fold into rG4 *in vitro* seem to be unfolded within the cell (27). That being said, one cannot exclude their formation at the level of the nascent RNA. More straightforward approaches need to be implemented to better document FHD–RNA interactions within the cell, irrespective of whether they are folded as rG4s or not, such as RIP-ChIP, CLIP-seq (33) and thus to unravel a potential functional impact.

A recent paper has revealed an underappreciated role for DNA G4 in TF recruitment. This study discovered that endogenous G4s can be prevalent TF-binding sites in the human genome. Interestingly, endogenous G4s are binding sites for a large number of TFs, especially at promoters of highly expressed genes, functioning as TF-binding hubs. From a biochemical perspective, they showed that a series of TFs interacted with ssDNA able that fold into well-characterized G4s. Interestingly, they also found that FOXA1 was not enriched at DNA G4s (and was used as a negative control). In agreement with our own findings with FOXL2, they failed to detect FOXA1 binding to ssDNA (31). The explanation cannot be that the RF sequences as ssDNA cannot form G4 structures *in vitro* because a previously reported thrombin ssDNA aptamer has a closely related sequence and does form G quadruplexes (34,35).

Our results showing that TFs are able to interact with RNA lends credence to the TF-cycle hypothesis (36). In short, FOX factors bound to genomic domains engaged in transcription (functional binding (37)) or not (non-functional binding at storage sites? (37)) could swap from the DNA to nascent RNAs bearing G_2_-rich sequences during transcription. Pre-RNPs containing bound FOX factors would release them back upon processing to reinitiate new rounds of transcription. When the G_2_-rich sequences remains in the mature RNAs, FOX factors could also be transiently exported to the cytoplasm. Obviously, this possibility has to be considered in the context of the cell steady-state and does not necessarily imply a sudden nuclear depletion of FOX factors leading to a ‘universal’ misregulation of transcription. However, one can envisage that TF sequestration on RNA in the absence of processing could constitute a negative feedback loop able to alter the rate of transcription.

Guo and Bartel have found evidence against the *in vivo* folding of *in vitro*-forming rG4 regions. This suggests the existence of ATP-dependent molecular machines able to remodel rG4s and maintain them unfolded (27). This implies that decreasing the activity/expression of such proteins would increase the titrating capacity of folded rG4, which could have a functional impact on transcription. Finally, one cannot exclude the existence of allosteric effects induced by the interaction with rG4s that would modify the properties of the relevant TFs. In the context of nucleo-cytoplasmic traffic mediated by rG4-containing transcripts, the amount of FOXL2 exported is expected to be low because no shuttling has ever been reported for this TF. This is not the case for FOXO factors, which are known to shuttle between the nucleus and the cytoplasm (38).

As mentioned above, polyAla expansions of FOXL2 account for 30% of the reported intragenic variants. On molecular grounds, we have shown that this pathogenic variant leads to intranuclear aggregation and mislocalization of the protein owing to cytoplasmic aggregation/retention (6,39,40). In addition, polyA-expanded FOXL2 can retain the wt counterpart in aggregates (6). In the light of our results, we hypothesize that FOXL2 aggregates might also retain RNAs. In short, coaggregation would diminish not only the pool of active protein but also the pool of RNAs containing G_2_-rich sequences described here. This idea deserves further exploration.

In sum, to better understand the functional impact of the interactions between FOXL2 and ssRNA, solving the structure of the binary complex would be required to guide mutagenesis experiments to disentangle the involvement of FOXL2 in transcription from other RNA-related processes. This is a critical step because even subtle variations of the FHD of FOXL2 lead to protein aggregation (40).

## Supplementary Material

gkad994_Supplemental_Files

## Data Availability

The RNA-seq data underlying this article are available in Gene Expression Omnibus at https://www.ncbi.nlm.nih.gov/geo/ under the accession number GSE227040. All fastq files of SELEX are available in the European Nucleotide Archive (ENA) at https://www.ebi.ac.uk/ena/browser/home under accession code PRJEB62756. Further data underlying this article will be shared on reasonable request to the corresponding author.
