# Supplementary material for: The forkhead DNA-binding domain binds specific G2-rich RNA sequences
Source: Nucleic Acids Res. 2023 Nov 2;51(22):12367–80. doi: 10.1093/nar/gkad994 (PMC10711433; doi:10.1093/nar/gkad994)
Supplement: gkad994_Supplemental_Files [file gkad994_supplemental_files.zip › Supplementary Figures.pdf]

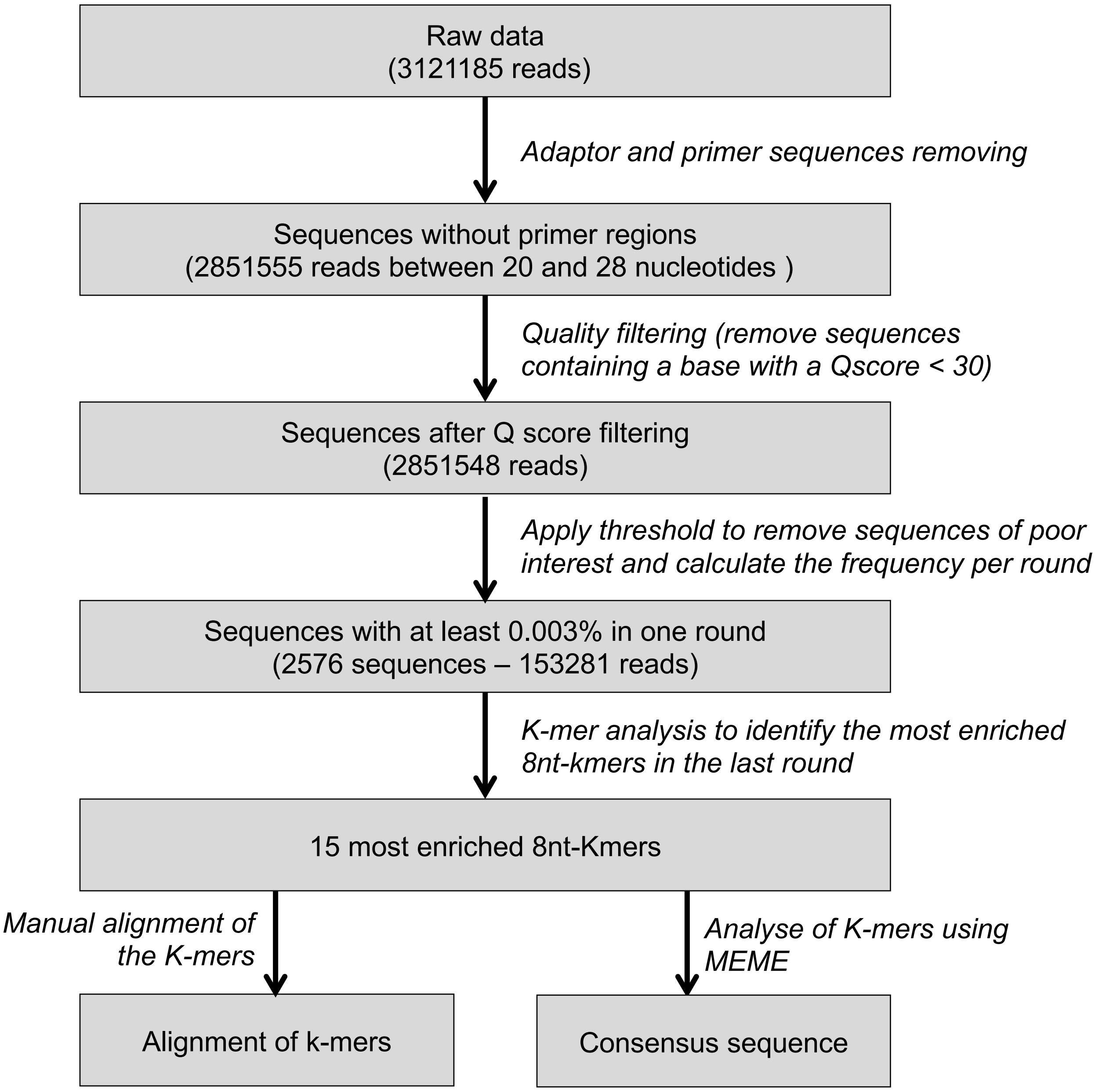

Supplementary Figure S1: Sequencing analysis workflow of DNA SELEX against wtFOXL2

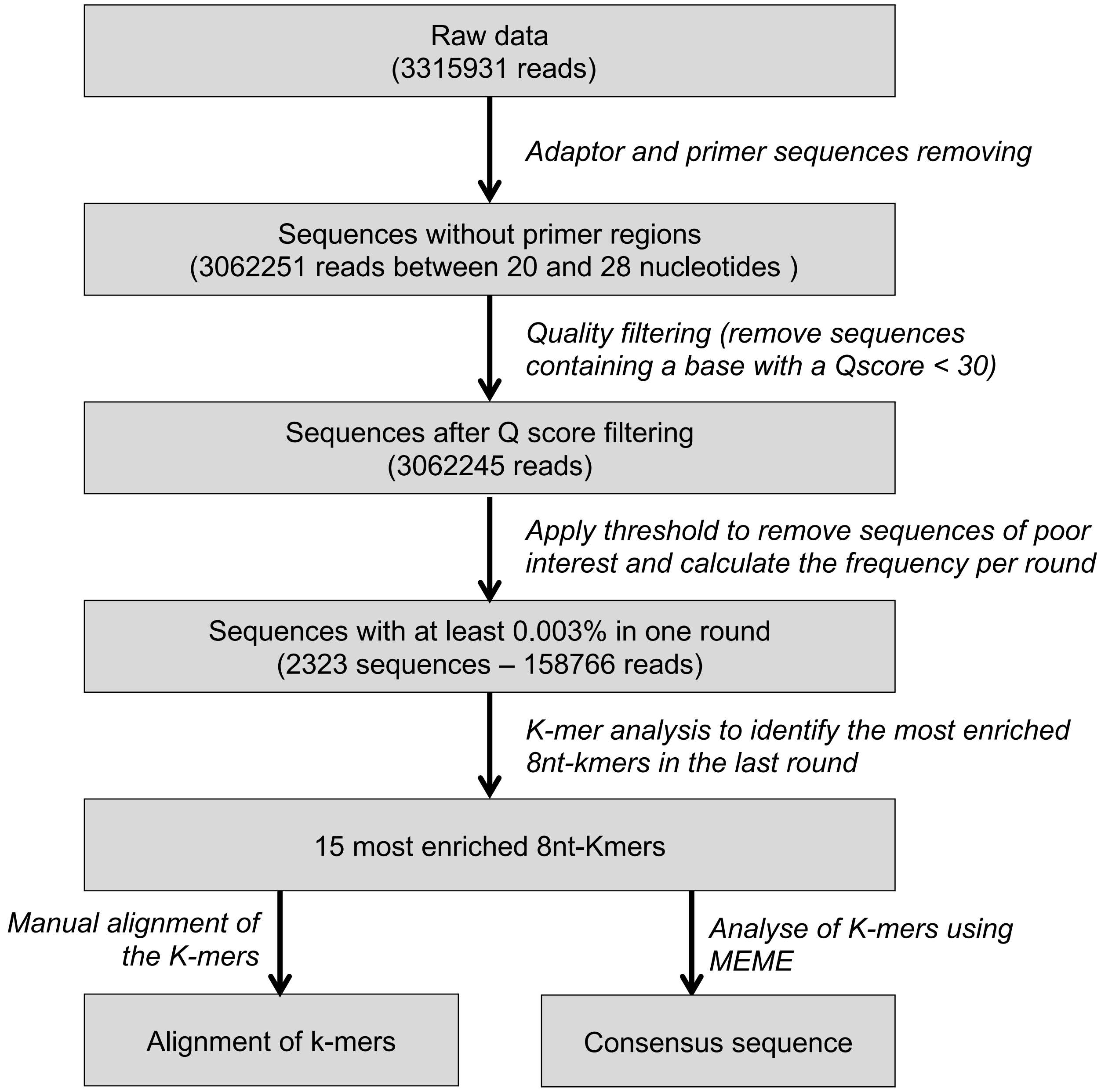

Supplementary Figure S2: Sequencing analysis workflow of DNA SELEX against mutFOXL2

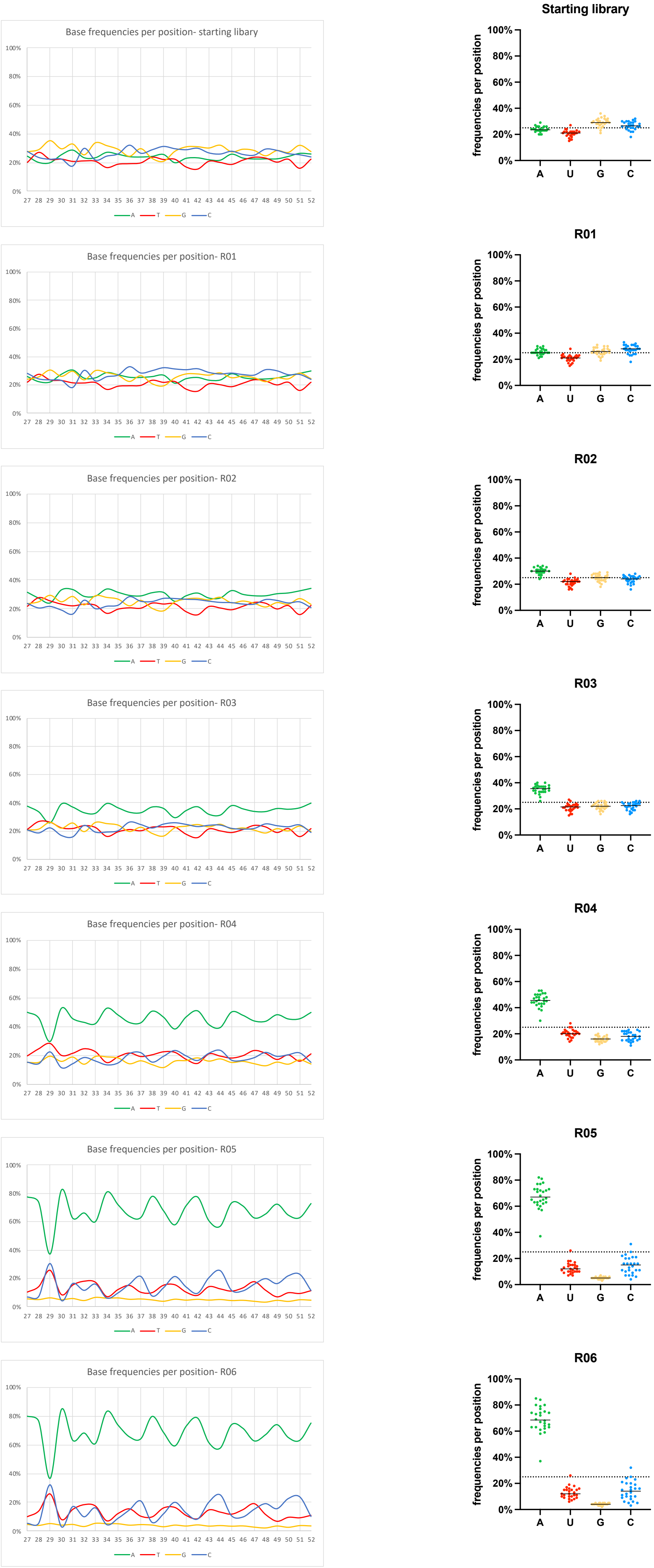

Supplementary Figure S3: Evolution of nucleotide frequency during DNA SELEX against wtFOXL2. The average frequency of each nucleotide at each position was calculated for libraries from different rounds of selection. The library after round 1 is still unbiased in terms of base composition, whereas A frequency increases over rounds.

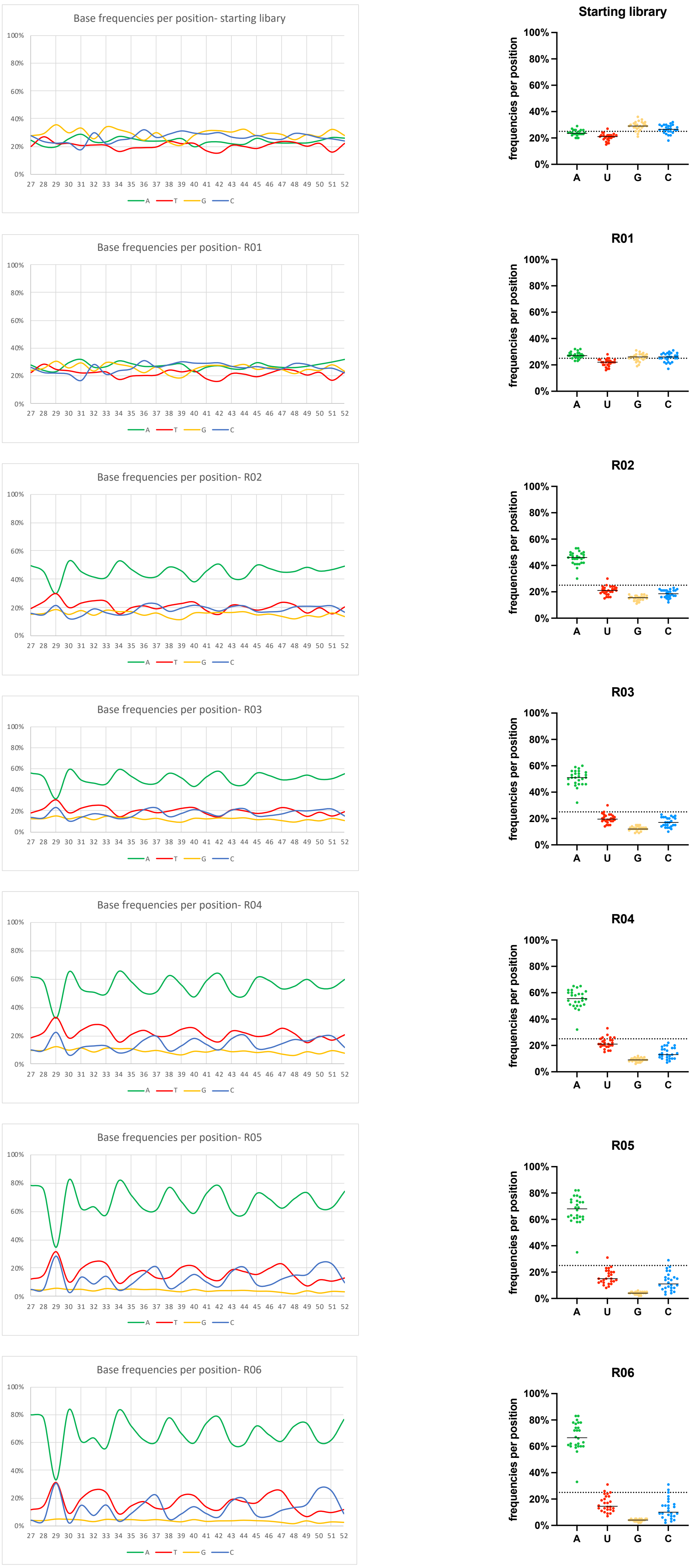

Supplementary Figure S4: Evolution of nucleotide frequency during DNA SELEX against mutFOXL2. The average frequency of each nucleotide at each position was calculated for libraries from different rounds of selection. The library after round 1 is still unbiased in terms of base composition, whereas A frequency increases over rounds.

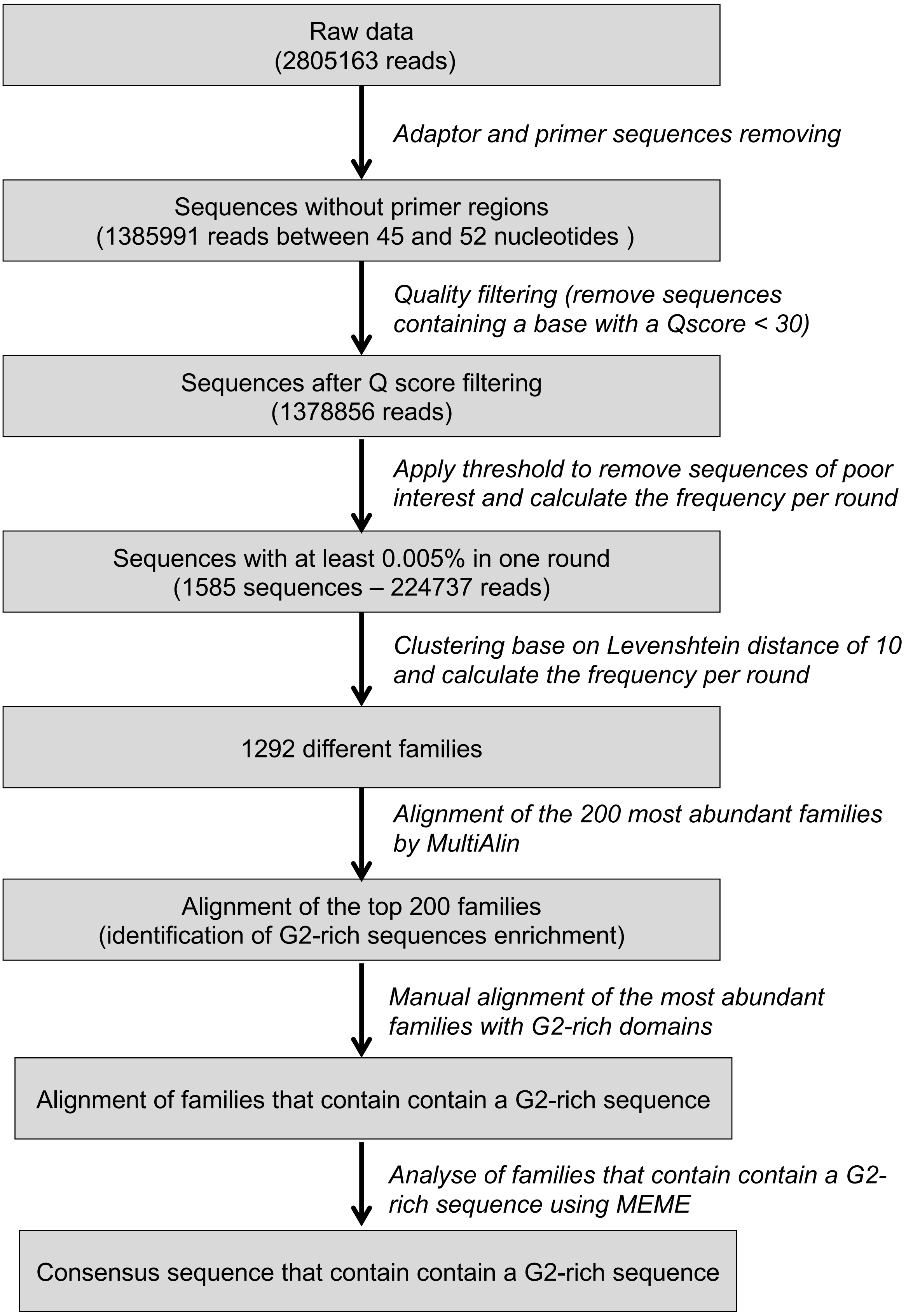

Supplementary Figure S5: Sequencing analysis workflow of RNA SELEX against wtFOXL2

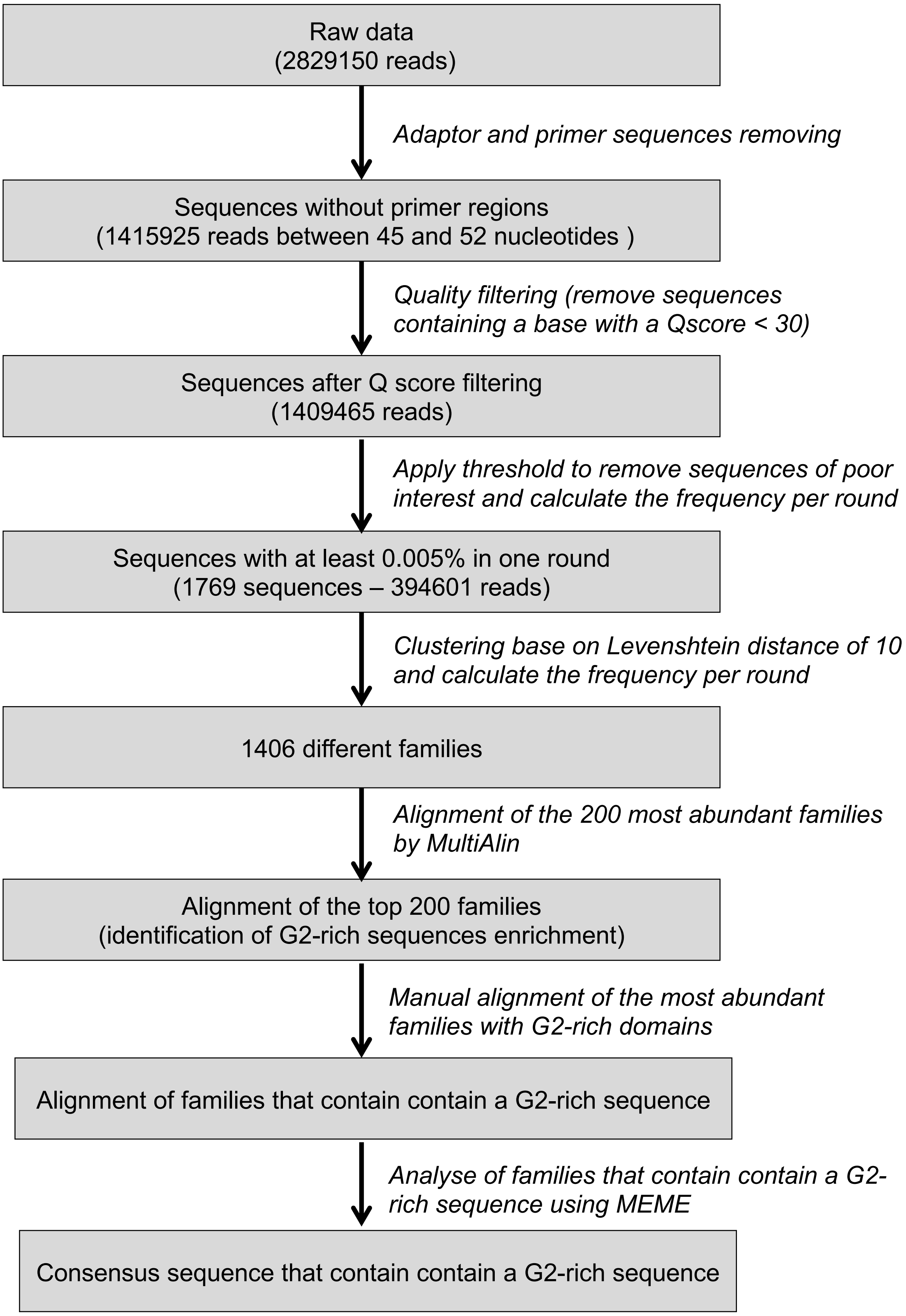

Supplementary Figure S6: Sequencing analysis workflow of RNA SELEX against mutFOXL2

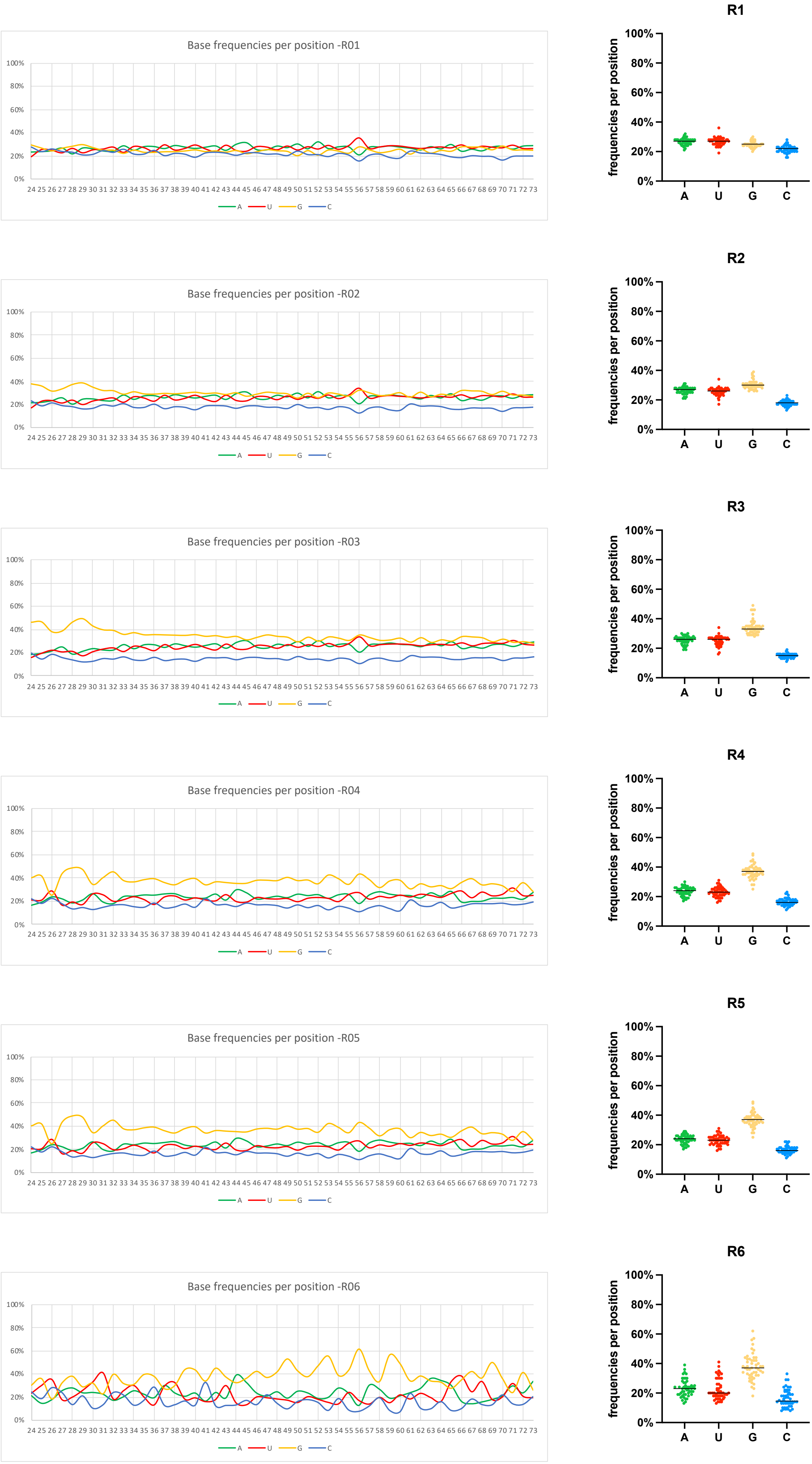

Supplementary Figure S7: Evolution of nucleotide frequency during RNA SELEX against wtFOXL2. The average frequency of each nucleotide at each position was calculated for libraries from different rounds of selection. The library after round 1 is still unbiased in terms of base composition, whereas G frequency increases over rounds, reflecting enrichment of G-rich sequences.

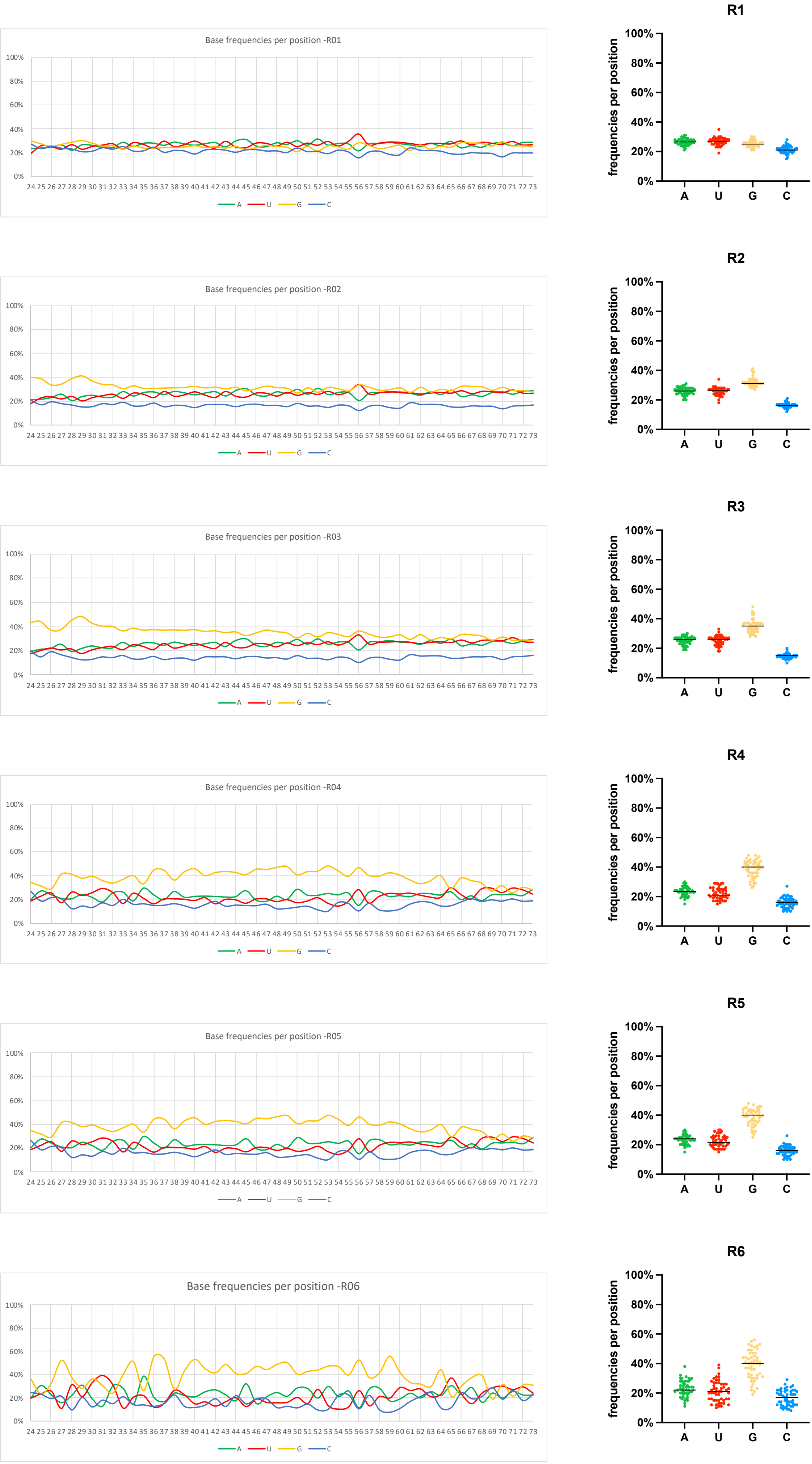

Supplementary Figure S8: Evolution of nucleotide frequency during RNA SELEX against mutFOXL2. The average frequency of each nucleotide at each position was calculated for libraries from different rounds of selection. The library after round 1 is still unbiased in terms of base composition, whereas G frequency increases over rounds, reflecting enrichment of G-rich sequences.
